# Encephalitozoon intestinalis: A Rare Cause of Diarrhea in an Allogeneic Hematopoietic Stem Cell Transplantation (HSCT) Recipient Complicated by Albendazole-Related Hepatotoxicity

**DOI:** 10.4274/Tjh.90692

**Published:** 2013-06-05

**Authors:** Serdar Şıvgın, Bülent Eser, Leylagül Kaynar, Fatih Kurnaz, Hülya Şıvgın, Süleyman Yazar, Mustafa Çetin, Ali Ünal

**Affiliations:** 1 Erciyes University, School of Medicine, Department of Hematology, Dedeman Stem Cell Transplantation Hospital, Kayseri, Turkey; 2 Erciyes University, School of Medicine, Department of Medical Genetics, Kayseri, Turkey; 3 Erciyes University, School of Medicine, Department of Parasitology, Kayseri, Turkey

**Keywords:** Allogeneic hematopoietic stem cell transplantation (allo-HSCT), Hepatotoxicity, Encephalitozoon intestinalis, Albendazole

## Abstract

A 50-year-old male patient previously diagnosed with acute myelomonocytic (M4) leukemia in July 2009 underwent allogeneic hematopoietic stem cell transplantation (allo-HSCT). During the pre-transplant period complete blood count (CBC), liver and renal function tests, coagulation tests, and other parameters were normal. On the first day of transplantation teicoplanin (400 mg d–1 for the first 3 d, and then 400 mg d-1) and caspofungin (first dose was 1×70 mg d–1, followed by 1×50 mg d–1) were started intravenously due to white plaques and oropharyngeal candidiasis in the patient’s mouth and perianal erythema. On the 14th d of transplantation watery diarrhea occurred, along with abdominal discomfort, nausea, and fatigue.

Stool examination was negative for findings of bleeding. Investigation of Microsporidia confirmed a rare pathogen Encephalitozoon intestinalis in the patient’s stool sample via species-specific immunofluorescence antibody (IFA) assay and albendazole treatment was started at a dose of 2×400 mg d–1. On the 5th d of albendazole treatment (d 18 of treatment) liver function test (LFT) results began to deteriorate. As LFT results continued to deteriorate, albendazole was withdrawn on the 7th d of treatment. Biopsy was performed on the 22nd d of transplantation and histopathological analysis confirmed the diagnosis of toxic hepatitis. LFT results began to decrease after withdrawal of albendazole treatment. On the 13th d of albendazole treatment all LFT values returned to normal. The presented allo-HSCT case had a rare pathogenic agent

(E. intestinalis) that caused diarrhea, as well as hepatotoxicity due to albendazole treatment. This is the first reported case of E. intestinalis diagnosed via IFA in Turkey.

**Conflict of interest:**None declared.

## Abstract (tr)

Önceden akut myelomonositik lösemi(M4) tanısı almış 50 yaşındaki erkek hastaya, Temmuz 2009’da allojenik hematopoietik kök hücre nakli yapıldı (AHKHN). Nakil öncesi dönemde, tam kan sayımı, karaciğer ve böbrek fonksiyon testleri, koagulasyon parametreleri ve diğer ölçümler normal bulundu. Naklin birinci gününde,orofaringeal candidiasisle birlikte ağız içindeki beyaz plaklar ve ayrıca perianal eritem nedeniyle hastaya intravenöz teikoplanin (ilk 3 gün 400mg/gün ve sonrasında günde 400mg) ve kaspofungin (ilk doz 1x70 mg/gün ve sonrasında 1x50 mg/gün) başlandı. Naklin 14.gününde,karında huzursuzluk, bulantı ve yorgunluk gibi şikayetlerle birlikte sulu diare ortaya çıktı. gaita incelemesinde kanama bulgusu yoktu. Tür-özgün IFA metodu ile nadir bir patojen olan Encephalitozoon intestinalis tesbiti doğrulandı ve 2x400 mg/gün albendazol tedavisi hemen başlandı. Albendazol tedavisinin 5. gününde (naklin 18. günü), hastanın karaciğer fonksiyon testleri (KCFT) bozulmaya başladı. KCFT’nin bozulması devam ettiğinden, tedavinin 7. gününde albendazol kesildi. Naklin 22. gününde Kc biopsisi yapılarak, tanı patologlar tarafından ‘toxik hepatit’ olarak doğrulandı. KCFT albendazol tedavisinin kesilmesinden sonra hızla düzelmeye başladı. albendazol tedavi sürecinin 13.gününde tüm KCFT değerleri normale döndü. Bu vaka; AHKHN yapılmış bir hastada nadir diare etkeni - Encephalitozoon intestinalis- ile albendazol tedavisi sırasında gelişen hepatotoksisiteyi göstermektedir. Ayrıca, IFA metodu ile Türkiye’den bildirilen ilk E. intestinalis vakasıdır.

## INTRODUCTION

Diarrhea is a major cause of morbidity and discomfort in patients undergoing high-dose chemotherapy and allogeneic hematopoietic stem cell transplantation (allo-HSCT) [1]. Infectious events, such as bacterial and viral gastro-enteritis, may be a more frequent cause of diarrhea than previously thought [2]. Inflammation of the intestinal mucosa due to chemotherapy, use of multiple medications such as prophylactic antimicrobials, and infection are common causes of diarrhea in allo-HSCT patients [3]. The incidence of infectious gastro-enteritis associated with allo-HSCT and autologous HSCT varies from 13% to 40% [4].

Microsporidia are obligate intracellular parasites that are recognized as important opportunistic pathogens in immunocompromised and transplanted patients [4,5]. Enterocytozoon bieneusi and, less frequently, Encephalitozoon intestinalis are the most prevalent Microsporidia species in humans; both of these are associated with enteric infections. In clinical practice, albendazole is widely used for treatment of these pathogens. Herein we present a case previously diagnosed as myelomonocytic leukemia that underwent allo-HSCT and was complicated by hepatotoxicity due to antimicrobial treatment for a rare pathogenic microorganism E. intestinalis.

## CASE

A 50-year-old male patient previously diagnosed with acute myelomonocytic (M4) leukemia in July 2009 was given 2 courses of doxorubicin and cytarabine as induction chemotherapy. After complete remission was achieved, high-dose cytarabine was given as a consolidation regimen. The patient had 1 mismatched donor and underwent allo-HSCT. The conditioning regimen was busulfan + cyclophosphamide. The laboratory findings were as follows: HBsAg: (–); anti-HDV: (–); anti-HCV: (–); anti-HEV: (–); anti-HBs: 96 mIU mL–1; anti-HBe: (–); ant-HBc Ig M: (–); anti-CMV Ig M: (–); anti-EBV Ig M: (–); anti-ParvoV IgM: (–); anti-HIV: (–). Clostridium difficile toxin A was negative and genetic analysis for inv (16) was negative. Complete blood count analysis was as follows: Hb: 14.6 dL–1 (13-17 g dL–1); WBC: 8790 µL–1 (4-10×103 μL–1); platelet count: 271,000 µL–1 (150-400×103 μL–1). Biochemical findings were as follows: glucose: 69 mg dL–1 (74-106 mg dL–1); BUN: 20 mg dL–1 (7.9-21 mg dL–1); creatinine: 0.87 mg L–1 (0.66-1.09 mg L–1); Ca: 10.3 mg dL–1 (8.8-10.6 mg dL–1); ALT: 20 u L–1 (0-35 u L–1); AST: 30 u L–1 (0-31 u L–1); ALP: 83 u L–1 (30-120 u L–1); GGT: 25 (0-38); albumin: 4.9 d L–1 (3.5-5.2 g dL–1); direct/indirect bilirubin: 1.01/0.19 mg dL–1 (0.3-1.2/0-0.2 mg dL: –1); LDH: 230 u L–1 (100-245 u L–1). Prothrombin and activated partial thromboplastin time was 12 and 40.8 s–1 (12-18 and 26-44 s–1), respectively. 

During the pre-transplant period the patient’s complete blood count (CBC), liver and renal function tests, coagulation tests, and other parameters were normal. No genetic aberrations or mutations were observed. Following administration of the conditioning regimen (busulphan + cyclophosphamide), the patient underwent allo-HSCT in January 2010. On the first d of transplantation teicoplanin (400 mg d–1 for the first 3 d, and then 400 mg d–1) and caspofungin (first dose 1×70 mg d–1, followed by 1×50 mg d–1) were given intravenously due to white plaques and oropharyngeal candidiasis in the patient’s mouth, and perianal erythema. On the second d of transplantation, imipenem (4×500 mg d–1) was added to the treatment due to neutropenic fever.

**Figure 1:**
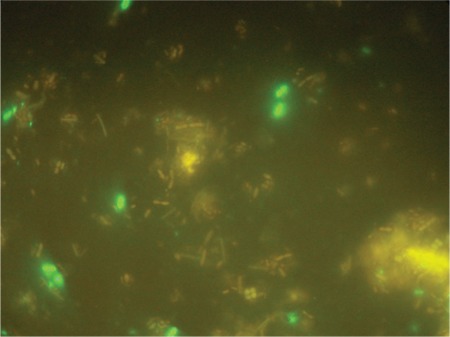
E. intestinalis spores with IFA method.

On the 14th d of the transplantation watery diarrhea occurred, along with abdominal discomfort, nausea, and fatigue. Stool examination showed no findings of bleeding, but a rare pathogen E. intestinalis was detected via immunofluorescence antibody (IFA) assay (Figure 1), which was performed according to the manufacturer’s instructions, as follows: stool sample was diluted with PBS and filtrated through a 50-μm filter; 2 μL of the fecal sample suspension was placed on 18-well slides and dried for 1 h; the slides were fixed with methanol dipped subsequently in acetone for 10 min at –20 °C; 20 μL of the monoclonal antibodies was added to the slides and incubated for 30 min at room temperature in a humid atmosphere; the slides were washed in PBS 3 times; 20 μL of conjugate was added to the slides and incubated for 30 min at room temperature in the dark; cover slips were mounted on glass slides with 3 drops of anti-fading fluorescence mounting medium and viewed with a fluorescence microscope equipped with the 450-nm fluorescein filter.

Immediately after the diagnosis was confirmed, albendazole treatment was started at a dose of 2×400 mg d–1. On the fifth d of albendazole treatment (18th day of treatment) LFT results began to deteriorate. First, teicoplanin was discontinued on the 18th day of transplantation, and imipenem together with caspofungin was initiated on the 19th d. As the LFT results continued to deteriorate, albendazole was discontinued on the seventh d of albendazole treatment (Table 1). The patient was thought to have toxic hepatitis and percutaneous liver biopsy was performed for differential diagnosis. During the pre-transplant period the patient did not have liver function abnormality and abdominal ultrasonography showed no pathological findings, such as hepatosteatosis or cholestatic disorder.

**Figure 2:**
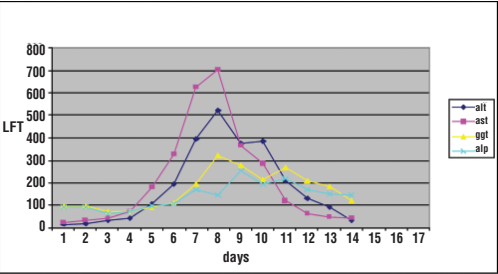
LFT after albendazole treatment.

After the biopsy was performed on the 22nd day of transplantation, histopathological analysis of the specimen confirmed the diagnosis of toxic hepatitis. Stool examination was repeated 8 d after the diagnosis and was negative. As the patient’s diarrhea resolved, the medications used to treat diarrhea were withdrawn. LFT results began to decrease immediately after the discontinuation of albendazole treatment. On the 13th d of albendazole treatment, all LFT values returned to normal (Figure 2). The patient was discharged on d 34 of treatment.

## DISCUSSION

Patients that undergo allo-HSCT are at risk of severe infectious complications during the post-transplant period. Early infections are primarily attributed to neutropenia and to microbial invasion due to the breakdown of mucosal barriers. Microsporidia can cause disease in human immunodeficiency virus-infected patients and other immunocompromised individuals [6]. E. bieneusi and 

E. intestinalis are the 2 species that most often cause intestinal infection in humans, manifesting with malabsorption, watery diarrhea, abdominal pain, weight loss, and nausea. Serological studies have suggested that several species of Microsporidia may commonly infect immunocompetent adults; such infection is usually treated with albendazole and fumagillin.

The diagnosis of microsporidiosis is difficult. Although a variety of methods are used to detect Microsporidia in clinical laboratories, special stains and light microscopy are routinely used for detection [7,8]. Traditional PCR enhances the detection of Microsporidia and has been used in some clinical laboratories; however, many of the assays were developed for research purposes and do not lend themselves to routine use in clinical laboratories because of their cumbersome specimen processing and DNA extraction methods, as well as false-positive results [9], which may be due to cross contamination, and false-negative results that occur because of inadequate specimen storage, low target concentration, or the presence of fecal inhibitors; these include complex acidic polysaccharides protein, DNases, heme compounds, fat, and proteinases [10]. 

Liver dysfunction [11] is a common problem in HSCT recipients and it is important to determine its etiology in order to administer appropriate therapy. Liver dysfunction following HSCT may be due to a variety of causes, including graft-versus-host disease (GVHD), infections, hepatic veno-occlusive disease (VOD) drugs, transfusion-related hemochromatosis, viral hepatitis, and infiltration with leukemic cells [11,12]. In the presented patient there were no post-allo-HSCT complications due to the pre-transplant medication regimen. Anti-microbial prophylaxis consisted of oral fluconazole, moxifloxacin, acyclovir, and metronidazole, which were administered from the beginning of conditioning treatment until engraftment day (d 12). When the patient’s LFT results began to rise, we first considered that the other antiviral and antibiotic agents were the major cause of deterioration because there were no data available regarding the hepatotoxic effect of albendazole in allo-HSCT patients; however, albendazole treatment should be considered carefully in cases of hepatic dysfunction or chronic liver disease [13]. In the presented patient pre-transplant administration of metronidazole was not sufficient to avoid diarrhea due to E. intestinalis. This species is primarily diagnosed in patients that have undergone solid organ transplantation, but is very rare in allo-HSCT patients, and data must thus be carefully monitored for future advances. Current reports of the efficacy of albendazole in the treatment of AIDS patients with E. intestinalis infection are inconsistent. The clinical manifestations of E. intestinalis infection in immunocompetent patients range from asymptomatic infections to self-limited diarrhea. The infectious form is a spore (1.8-5.0 µm) that is very resistant to environmental conditions [14]. The detection of spores in human feces or other human bodily fluids is very cumbersome and difficult. Treatment of E. intestinalis infection should continue for 14 d. 

Differences in drug susceptibility between the various Microsporidia species that infect patients with AIDS have been documented in both prospective studies involving small numbers of patients and anecdotal case reports [15,16,17]. The most common clinical symptom of E. intestinalis infection is watery diarrhea [18], but symptoms indicative of dissemination to the urinary, hepatobiliary, and respiratory tracts are not unusual [19]. Preliminary data suggest that albendazole has potent antiparasitic efficacy in AIDS patients infected with E. intestinalis; however, experience with this drug is limited, as there have been fewer than 40 reported cases of human infection due to E. intestinalis [20]. To the best of our knowledge hepatotoxicity is not a rare complication of albendazole treatment, but the presented case is the first allo-HSCT patient to be diagnosed with 

E. intestinalis. The most common side effects of albendazole treatment are abdominal pain, nausea, vomiting, serum transaminase elevation, and rarely, leucopenia.

Prognosis after allo-HSCT is largely dependent on whether or not complications involving the liver, lung, and intestine can be avoided. This may be very important, as the diagnosis can be accomplished by interpretation of the clinical setting in which liver dysfunction occurs. As a conclusion, >1 possible cause of liver dysfunction may be identifiable in these critical patients, which can delay accurate diagnosis of any complication in the post-transplant period. In conclusion, pre-transplant prophylactic regimens may be insufficient to avoid all complications during the post-transplant period, and a wide variety of possibilities should be taken into account while evaluating clinical and laboratory abnormalities. The presented case is the first to have E. intestinalis infection diagnosed via IFA in Turkey. This case presentation is the first to describe an allo-HSCT patient with a rare pathogenic agent E. intestinalis that caused diarrhea, as was well as hepatotoxicity due to albendazole treatment.

## CONFLICT OF INTEREST STATEMENT

The authors of this paper have no conflicts of interest, including specific financial interests, relationships, and/or affiliations relevant to the subject matter or materials included. 

## Figures and Tables

**Table 1:**
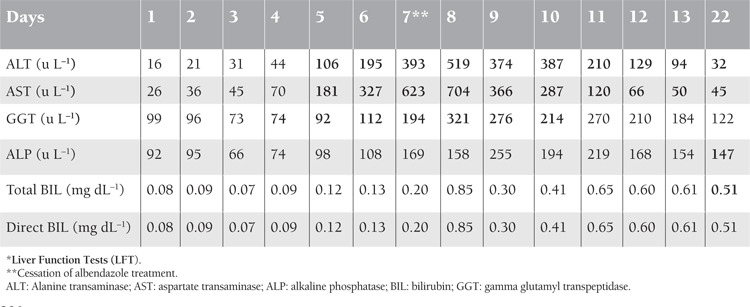
*LFT after albendazole treatment.
